# Spin-state switching of indium-phthalocyanine on Pb(100)†

**DOI:** 10.1039/d4ra07270g

**Published:** 2024-12-05

**Authors:** Niklas Ide, Arnab Banerjee, Alexander Weismann, Richard Berndt

**Affiliations:** a Institut für Experimentelle und Angewandte Physik, Christian-Albrechts-Universität zu Kiel D-24098 Kiel Germany berndt@physik.uni-kiel.de

## Abstract

Indium(iii) phthalocyanine chloride deposited on Pb(100) is studied by scanning tunnelling spectroscopy at cryogenic temperatures. The Cl ions are dissociated and the remaining indium phthalocyanine (InPc) is observed in two states with the metal ion pointing to (↓) or away (↑) from the substrate. Isolated molecules and islands with a 
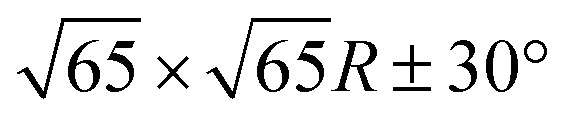
 superstructure and a unit cell of four inequivalent molecules, namely one InPc↑ and three InPc↓ in different sites, are observed. Using atomic resolution images of the substrate the adsorption sites and azimuthal orientation of InPc are determined and a structure model is proposed. Conductance spectra of the lowest unoccupied molecular orbital reveal differences that depend on the adsorption sites and azimuthal orientations of the complexes. Only InPc↑ molecules exhibit Shiba states, indicating the presence of a localized spin. By electron extraction isolated complexes as well as molecules in islands are converted from InPc↑ to InPc↓. At the same time, their spin state changes, as indicated by the disappearance of the Shiba states.

## Abstract 2

Spin state transition in an artificial array of indium phthalocyanine molecules. Scanning tunneling microscopy images show a 3 × 3 array before and after inducing a transition of the central molecule.

## Graphical abstract

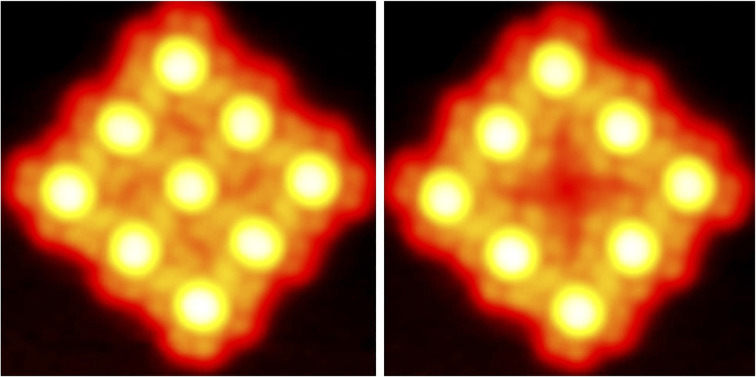

## 1 Introduction

Electrical control over the spin states of adsorbed molecules appears attractive for molecular spintronics devices. Spin switching of organometallic complexes is usually achieved by modifying their ligand shell. In a few cases, the controlled spin state switching of single molecules on surfaces has been reported. The complexes used predominantly involved Fe^II^ ions and N-based ligands.^1–11^ Fe^III^,^12^ Co,^13^ and Ni^14,15^ centres have occasionally been employed as well.

**Fig. 1:**
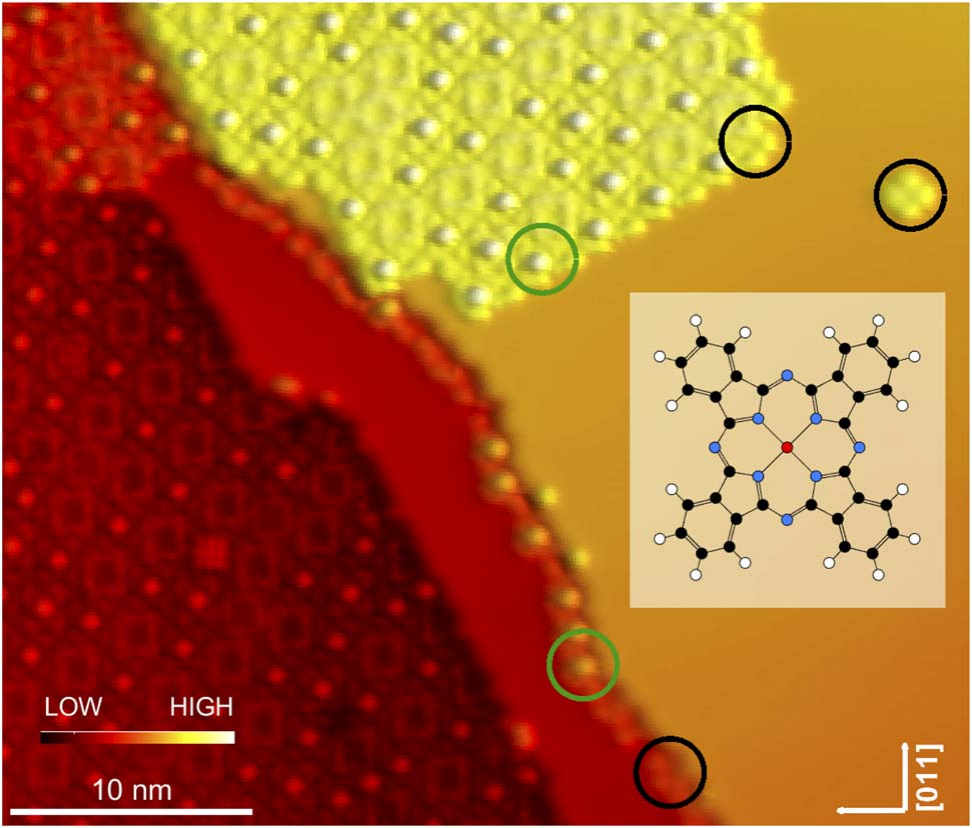
Topograph of a submonolayer coverage of InPc on Pb(100) (*V* = 200 mV). The overview image covers three Pb terraces, which are separated by two monatomic steps. The molecules exhibit either a central depression or a protrusion. A few examples are indicated by black and green circles, respectively. The insert shows a schematic of InPc. White, black, blue, red dots represent hydrogen, carbon, nitrogen, and indium, respectively.

Here, we present an attempt to harness a different class of molecules for spin switching, namely non-planar phthalocyanines, in particular In phthalocyanine (InPc) as shown in the insert of Fig. 1. As observed from SnPc^16–20^ and PbPc^21–23^ the non-planar complexes can adsorb with the metal ion pointing to (↓) or away (↑) from the substrate. For SnPc on Ag(111), conversion from ↑ to ↓ has been achieved by manipulation with the tip of a scanning tunnelling microscope (STM). While the transition was irreversible for molecules adsorbed directly on the Ag(111) substrate used bidirectional switching was possible for molecules in higher layers.^20,24^

In the case of InPc studied here, we similarly find current-induced switching from InPc↑ to InPc↓. By using a superconducting Pb(100) substrate, we obtain sensitivity of conventional scanning tunnelling spectroscopy to the spin state of the molecule. The ↑ state of complexes in islands gives rise to peaks at energies ±*ε* in the excitation gap of the superconductor because Yu-Shiba-Rusinov (YSR) states^25–27^ occur as previously reported from adsorbed atoms and molecules.^22,23,28–40^ They reveal the presence of a localized spin. In contrast, the spectrum of molecules in the ↓ state is virtually identical to the substrate spectrum, indicating that this state is diamagnetic.

## 2 Methods

Experiments were performed with two scanning tunnelling microscopes operated in ultra-high vacuum at temperatures between 1.5 and 4.2 K. Pb(100) single crystals were prepared by cycles of Ar^+^ bombardment and annealing to ≈230 °C. STM tips were cut from a Pb wire and sputtered in ultra-high vacuum. ClInPc molecules were sublimated from a Knudsen cell onto the Pb surface at ambient temperature. For imaging, constant currents between *I* = 50 and 200 pA were chosen. In this range, no significant effect on the image contrast was observed, except for a global change of the tip height.

Phase sensitive detection was used to measure spectra of the differential conductance d*I*/d*V* at fixed tip position. To this end, sinusoidal modulations were added to the sample voltage *V* with amplitudes of 10 mV_PP_ and 60 μV_PP_ for overview spectra and spectra of the superconducting gap, respectively.

Switching experiments were carried out by placing the STM tip above the centre of an InPc↑ molecule at *V* = 500 mV and *I* = 100 pA. These parameters usually enabled stable imaging. Next, the tip position was frozen, *V* was abruptly changed to −500 mV and then slowly lowered to −3.1 V. The tunnelling current was monitored during this procedure. A sudden current drop indicated a transition from InPc↑ to InPc↓ as subsequently verified by imaging at *V* = 500 mV.

Density functional theory (DFT) as implemented in Gaussian 16W^41^ served to optimize the geometry of neutral and positively charged InPc molecules. The B3LYP functional^42–45^ and LanL2DZ basis sets^46–48^ were used.

## 3 Geometric structure

After deposition of a submonolayer amount of InPc at ambient temperature, a large majority of molecules assembles into islands with a square lattice (Fig. 1). In addition, some molecules are attached to the steps and very few isolated molecules (a single one in Fig. 1) are observed on terraces. Closer inspection reveals that a majority of molecules (examples indicated by black circles) displays a central depression while a protrusion is observed from others (green circles). We attribute the different image contrasts to InPc molecules whose In ion is below (InPc↓) or above (InPc↑) the molecular plane. In islands, the abundance ratio between down and up is ≈3 : 1. A similar ratio was found at steps. All isolated molecules observed after deposition, however, were InPc↓.

**Fig. 2:**
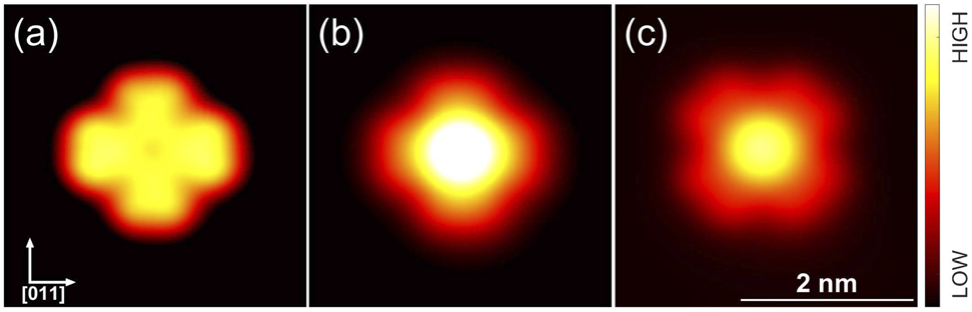
Topographs of isolated InPc complexes (*V* = 500 mV). The same colour scheme is used in all panels. (a) The molecular image displays a central depression. Four lobes are resolved and are oriented parallel to 〈011〉-directions of the substrate. Height range: 190 pm. (b) The molecule exhibits a central protrusion. The orientation of the lobes is identical to case (a). Height range: 290 pm. (c) A similar protrusion as in (b) is observed but the lobes are rotated by 45°. Height range: 330 pm.

We discard the possibility that the topographs in Fig. 2 shows undissociated ClInPc complexes that still carry their Cl ion. To verify the absence of Cl we heated the sample to ≈200 °C before imaging again at cryogenic temperature. We found no changes of the appearance of the complexes. In similar cases, ClAlPc^49^ and ClGaPc^50^ on Pb(100), heating to 200 °C leads to the desorption of Cl, presumably because of a surface-*trans*-effect.^51^ In addition, we performed manipulation experiments with InPc↑ complexes (see below). The data were consistent with a conversion from ↑ to ↓ and did not indicate any transfer of Cl to the tip as previously reported for FePc.^52,53^

Below, we first present detailed images of isolated molecules and then analyse the pattern found in self-assembled arrays.

### 3.1 Observed types of molecules

Fig. 2 shows constant-current topographs of isolated molecules. The image in Fig. 2(a) displays an as-deposited molecule. It exhibits four lobes oriented parallel to 〈011〉-directions of the substrate and a central depression. The molecules presented in panels (b) and (c) were prepared by moving molecules with a central protrusion away from a molecular island or a step edge with the STM tip. On the terrace, lacking neighbours, these molecules orientate their lobes either parallel or at an angle of 45° to a 〈011〉-direction (Fig. 2(b) and (c)).

### 3.2 Structure of ordered islands

**Fig. 3:**
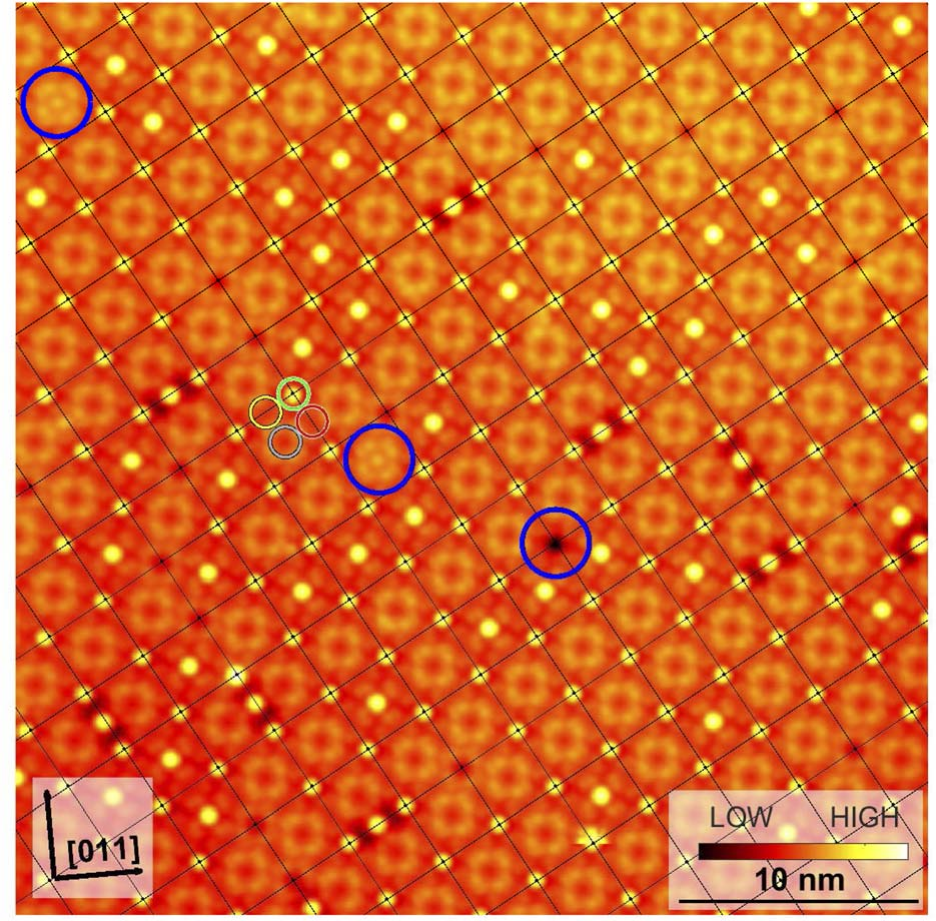
Topograph (*V* = 500 mV) of a monolayer island with a square superstructure. The overlayer mesh is indicated by black lines. Its is rotated by ≈30° compared to the Pb surface mesh. The unit cell contains four InPc molecules, at a corner (green circle), the centre (gray) and two edges (yellow, red). Three unknown molecules are found marked by blue circles.

Fig. 3 shows a typical sub-area from a monolayer island. Despite a degree of disorder a regular pattern is clearly discernible. The square overlayer mesh (lattice parameter ≈2.8 nm) is indicated by black lines. It is rotated by 30° compared to the Pb surface mesh. Islands with a −30° rotation (not shown) are also found on the surface as expected. The unit cell contains four InPc molecules as indicated in Fig. 3 by coloured circles.

**Fig. 4:**
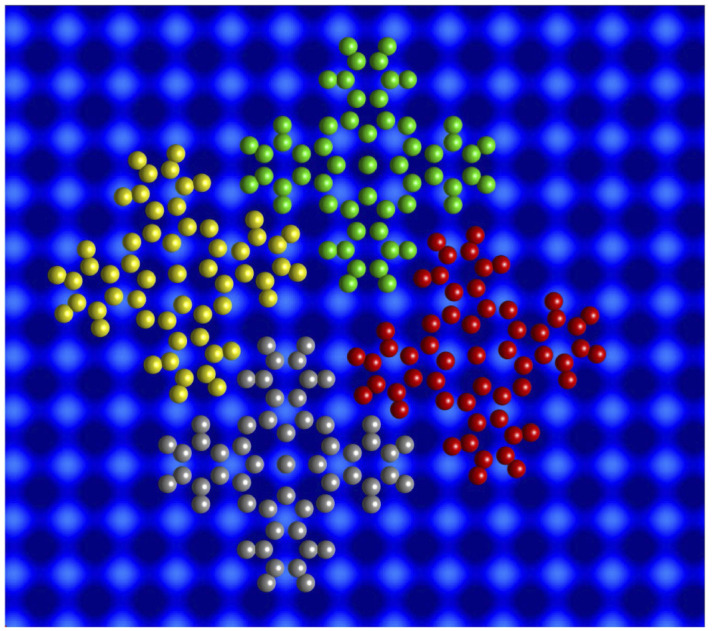
Model of the molecular unit cell in islands. The Pb(100) surface is shown by blurred blue spheres. The atoms of the InPc complexes are displayed using yellow, red, green, and gray spheres. While the green and gray molecules are centred at hollow and top sites of the Pb lattice the yellow and red molecules reside above bridge positions. These latter sites bridge atoms along the horizontal (*x*) and vertical (*y*) directions. The green molecules are InPc↑, all others are InPc↓. In addition to the structure shown, a mirror symmetric cell may be constructed.

Occasionally, atomic resolution imaging of the substrate close to islands was achieved. From such images (ESI†) the superstructure may be described as 
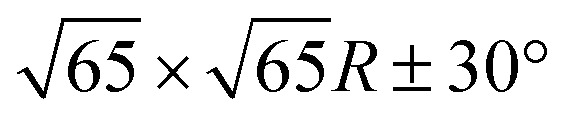
 or 
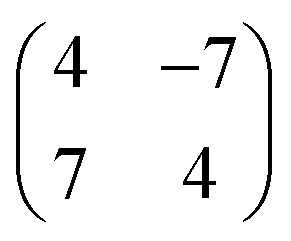
. In addition, the adsorption sites (defined by the position of the In ion) were determined. The resulting model is shown in Fig. 4. The complexes occupy top (grey), hollow (green), and bridge (red and yellow) sites. The lobes of the molecules in four-fold hollow and top sites are parallel to the high-symmetry substrate directions as observed from isolated molecules on terraces. The lobes of bridge-site molecules, however, are azimuthally rotated by 10°. We hint that this orientation reduces the overlap between the peripheral H atoms of nearest neighbours.

The model reproduces the 1 : 3 ratio of ↑ to ↓ found in ordered islands (*e.g.*, Fig. 3). Molecules at hollow sites are usually in the ↑ state while all other binding sites are most likely occupied by InPc↓ complexes. In addition to the ordered pattern, Fig. 3 some disorder is present. At top sites, less than one out 10 molecules is in the ↑ state rather than ↓. At hollow positions, some 30% of the complexes are InPc↓ rather than ↑. On the bridge sites, only ≈2.5% of the molecules are InPc↑ instead of ↓. Finally, less than 0.5% of the molecules could not be identified. We cannot exclude that kinetic limitations may affect these values despite the slow cooling to cryogenic temperatures over a period of ≈2 h. However, the rather different percentages of defects still indicate that bridge sites are particularly unfavourable compared to hollow sites.

## 4 Electronic structure

### 4.1 Spectroscopy of molecular orbitals

**Fig. 5:**
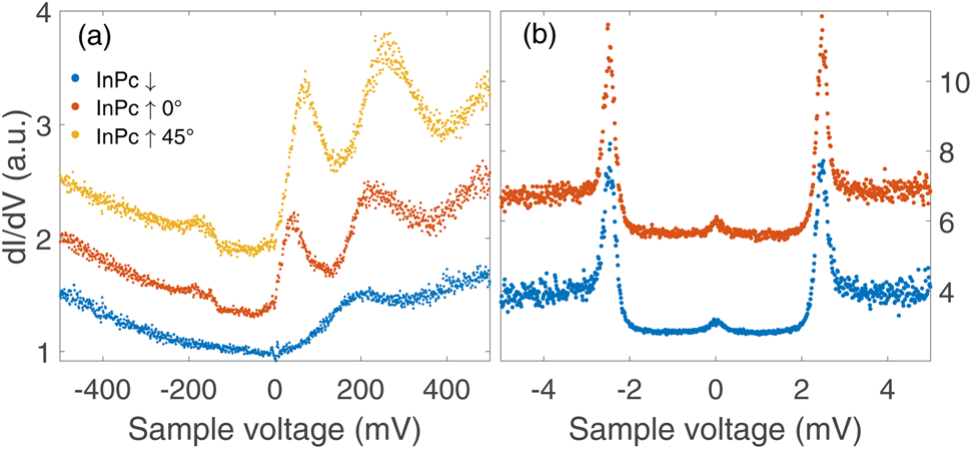
d*I*/d*V*-spectra recorded above the centre of isolated InPc molecules. (a) Spectra of the frontier orbitals of the observed configurations of InPc on Pb(100). Parameters used prior to freezing the tip position: *V* = 500 mV and *I* = 200 pA. (b) High resolution spectra of excitations close to the Fermi energy measured on the centre of isolated InPc↓ and InPc↑ (0° orientation) molecules. *V* = 6 mV, *I* = 200 pA. The spectra have been normalized to similar conductances at negative voltage and vertically shifted for clarity.

The three types of isolated InPc complexes exhibit distinct electronic states. Fig. 5(a) shows d*I*/d*V* spectra covering a fairly wide voltage range. On the InPc↓ complex (blue dots), a broad structure centred at ≈200 mV is observed on a parabolic background. The InPc↑ data (red for 45° orientation, yellow for 0°) exhibit more and significantly sharper features on a similar background. In both cases, a pair of excitations is resolved at positive sample voltage. The lower one is centred at approximately 35, 70, and 190 meV for ↑ 0°, ↑ 45°, ↓ molecules, respectively. We attribute it to the lowest unoccupied molecular orbital (LUMO). Due to its proximity to the Fermi level, it is most relevant for the magnetic properties of the complexes. The feature at higher energy is presumably a combined excitation of the LUMO and a vibration with an energy of ≈190 meV. The weak and broad structure discernible at negative voltages is tentatively attributed to vibrational excitations.

### 4.2 Spectroscopy of YSR states

Since the Pb tip of the STM is superconducting at the temperature of the experiment, its density of states (DOS) exhibits coherence peaks at the energies *Δ*_T_ and −*Δ*_T_. The DOS of the Pb sample has similar peaks at slightly different energies ±*Δ*_S_ and, in addition, may show YSR states at ±*ε*. Finally, the temperature is non-zero and consequently some thermally excited electrons and holes are present, in particular at the energies of the coherence peaks. The tunnelling pathways between these electrodes lead to peaks at ±(*Δ*_T_ + *Δ*_S_)/*e* (tunnelling between coherence peaks), ±(*Δ*_T_ − *Δ*_S_)/*e* (tunnelling of thermally excited electrons and holes), ±(*Δ*_T_ + *ε*)/*e* (tunnelling between a coherence peak and a YSR peak), and ±(*Δ*_T_ − *ε*)/*e* (tunnelling of excited carriers to or from a YSR peak).

Fig. 5(b) shows high resolution spectra of a ±5 mV range around the Fermi energy. The weak features detected around zero bias are due to the tunnelling of thermally excited electrons or holes. The dominant pair of peaks at ≈±2.5 mV observed for InPc↓ and InPc↑ as well matches the coherence peaks observed on pristine areas of Pb(100). No indication of a YSR state is discernible. Apparently, the occupation of the LUMO of both complexes is too low to give rise to a YSR state.

**Fig. 6:**
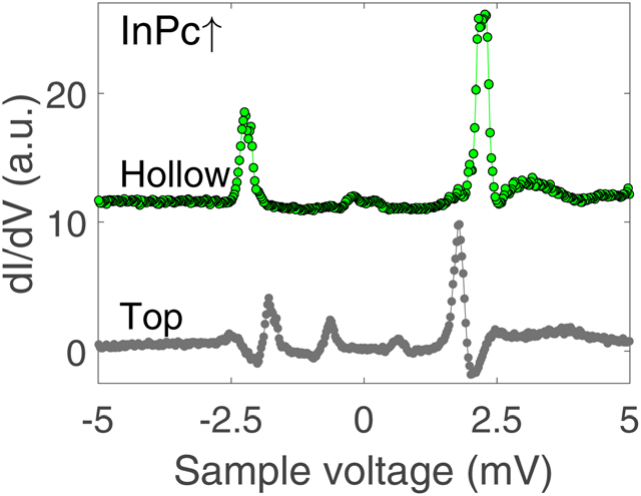
d*I*/d*V*-spectra recorded above the centre of InPc↑ molecules in ordered islands. Green and black dots show data from hollow and top site molecules, respectively. While clear YSR states are observed in both cases, the binding energies of the main states (green: 2.2 meV, black: 1.8 meV) are significantly different. Moreover, a second pair of peaks at ±2.5 mV is observed on the top site molecule. The features at ≈0.7 mV are due to tunnelling of thermally excited carriers and the YSR states (±(*Δ*_T_ − *ε*)/*e*).

In contrast to the isolated molecules, InPc↑ molecules in ordered islands show spectra with YSR states (Fig. 6). First, the dominant peaks occur at 2.2 and 1.8 mV at hollow and top sites, respectively, and thus are located in the superconductor gap. Second, a clear asymmetry with the bias polarity is observed. The coherence peaks at ±2.5 meV are discernible for the top site, which is not the case at hollow sites. Again, tunnelling of thermally excited charge carriers is found at low bias. As expected, these features occur at ≈0.7 (0.2) mV for the top (hollow) site. Although the data shown in Fig. 6 are typical, it should be noted that a survey of spectra from tens of molecules revealed further subtle variations. While it is clear that the variations are linked to the position of a given molecule in the lattice and with respect to defects a detailed interpretation is not yet available. InPc↓ molecules in islands do not exhibit YSR states.

## 5 Manipulation experiments and spin state switching

InPc molecules can be manipulated on Pb(100) by the STM tip. First, by decreasing the tip-sample distance, molecules may be removed from step edges and placed on open terraces. The current required to move InPc↓ molecules is larger than observed from InPc↑. Typical values used were *V* = 5 mV and *I* = 8 and 2 nA, respectively. This difference appears to be consistent with the stronger molecule–substrate coupling of InPc↓ suggested by the abundance ratio of the two orientations.

**Fig. 7:**
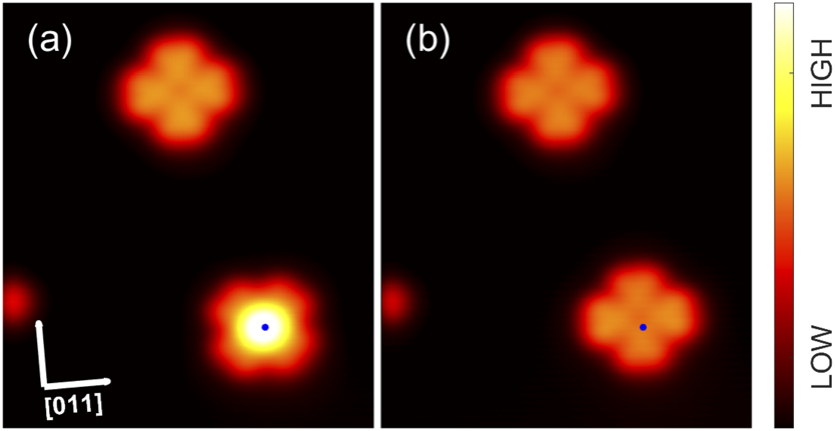
Manipulation of an isolated molecule. Topographs (*V* = 500 mV) showing two InPc molecules (a) before and (b) after applying *V* = −3.1 V to the ↑ molecule in the lower right corner. A small defect at the left edge of the image serves as a landmark. While the image of the upper molecule (↓) remains unchanged the lower molecule is converted from ↑ to ↓.

**Fig. 8:**
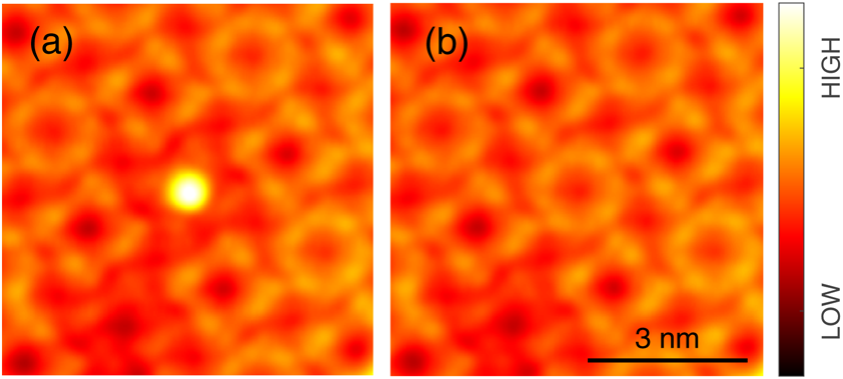
Manipulation of a molecule in an island. Topographs (*V* = 200 mV) of an InPc island (a) prior to (b) after applying an elevated voltage *V* = −3.1 V to an InPc↑ molecule at the centre of the image. After the manipulation, this molecule appears identical to the neighbouring InPc↓ complexes.

More importantly, InPc↑ complexes in arrays, which display a YSR feature and thus carry a spin, may be converted to spinless InPc↓. Fig. 7 and 8 show STM images recorded before and after such conversions. Fig. 7(a) displays two isolated molecules on a terrace. The lower molecule is a InPc↑ oriented at 45° that has been moved onto the terrace from a step edge. After applying slightly elevated current and voltage to the molecule, the InPc↓ state shown in panel (b) was obtained. In addition, the molecule has rotated to 0° orientation. In repeated experiments, we observed that ↑ to ↓ switching of isolated 45°↑ molecules usually led to a rotation to the preferred 0° state whereas 0° molecules never rotated to 45° orientation.

Data on the same kind of experiment performed with a InPc↑ molecule inside an island are presented in Fig. 8. Again, a transition from InPc↑ to InPc↓ is induced by applying suitable tunnelling parameters. Inside islands, rotation of the switched molecule was never observed as expected.

**Fig. 9:**
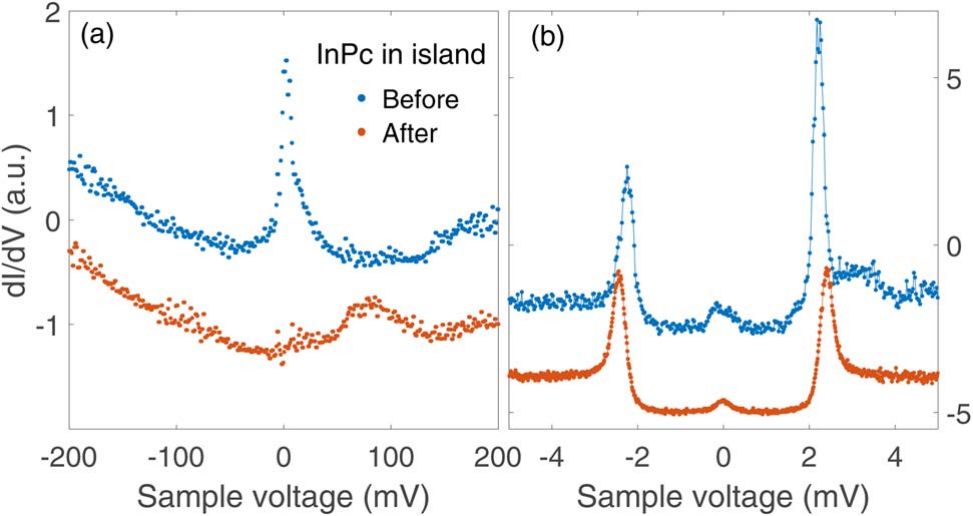
d*I*/d*V*-spectra recorded above the molecule manipulated as shown in Fig. 8. (a) Wide range spectra (set point 500 mV and 200 pA). The resonance at the Fermi energy observed from the pristine InPc↑ molecule (blue dots) is replaced by the LUMO peak near 80 mV, which is typical of InPc↓ with 0° orientation of the lobes. (b) Low energy spectra (6 mV, 200 pA). Before the manipulation, a small asymmetry of the heights of ±2.5 mV peaks signals YSR states close to the superconductor coherence peaks. After switching, the asymmetry is absent. All spectra are scaled to similar conductances at negative *V* and vertically shifted for clarity.

Spectra recorded above the centre of an InPc molecule before and after inducing a transition are shown in Fig. 9. In the wide bias range of Fig. 9(a), the molecule displays a sharp peak close to zero bias before the transition. After the transition, the peak is replaced by a broader maximum at higher *V*. We attribute both features to the LUMO of the corresponding molecule. Compared to isolated molecules, Fig. 5(a), the LUMO energies of InPc↑ and InPc↓ molecules in islands are shifted. The low energy spectra in Fig. 9(b) exhibit a clear peak height asymmetry before the transition which is absent afterwards. This reflects the presence and absence of a YSR state and is consistent with the observation that InPc↑ molecules in islands show a YSR state while InPc↓ do not.

Our DFT calculations of InPc in the gas phase suggest that the positively charged complex prefers a planar geometry while neutral InPc is distorted to a shuttlecock-like shape (ESI†). This finding is consistent with the STM images if we assume the transfer of an electron from the substrate to the complex in equilibrium. The current-induced transition then likely involves a planar transition state.

**Fig. 10:**
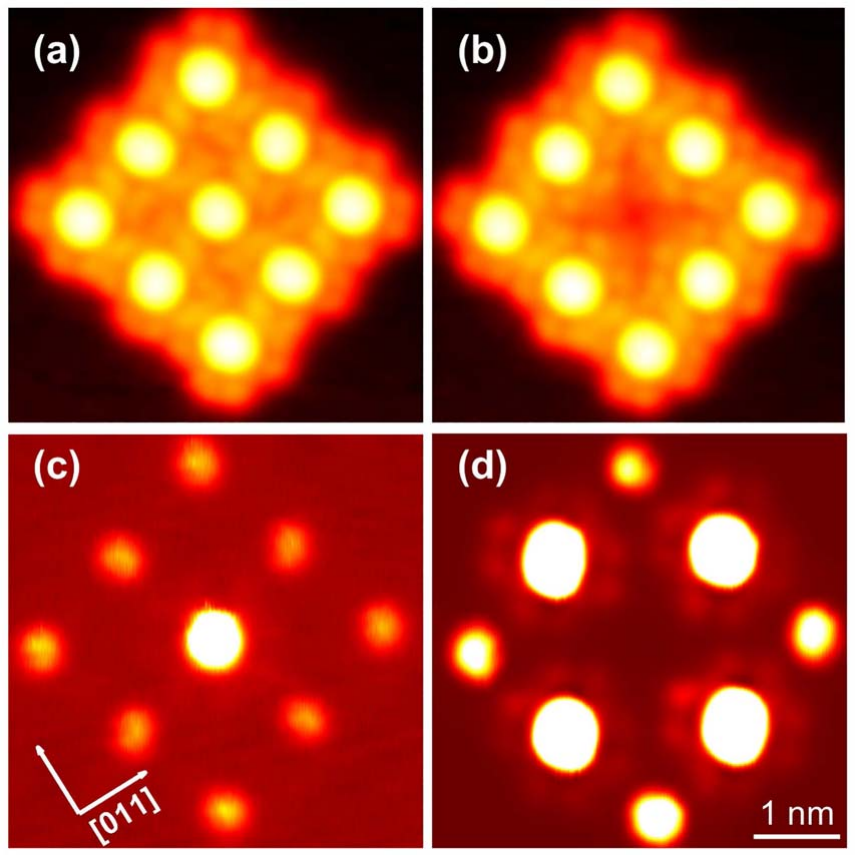
Spin state transition in an artificial array. (a) Topograph of a square 3 × 3 array of InPc↑ prepared by manipulation of molecules with the tip. All molecules were placed in top positions of the Pb mesh and a nearest neighbor distance of ≈1.4 nm was chosen. This arrangement is different from the structure obtained by deposition and annealing. (b) Topograph of the same cluster after the central molecule has been converted to InPc↓. (c and d) Maps of the current at *V* = 2 mV recorded at a constant height set at 3 mV and 100 pA from the same area as (a) and (b). The ranges covered by the colour scales of these maps have been separately adjusted for clarity.

By manipulation with the STM tip, artificial arrays can be constructed that correspond to other molecular arrangements than found in the islands analysed so far, which had been obtained by deposition at ambient temperature. Fig. 10(a) shows an example, namely nine molecules in a square array. All molecules are in the ↑ state unlike in the pattern in islands. Furthermore, the intermolecular distance (1.4 nm) and the azimuthal orientation of the square mesh (*R*14°) are different from the natural pattern. Next, by applying −3.1 V, the centre molecule was converted to its ↓ state as reflected by the contrast change in the image. Interestingly, this modification of the centre molecule leads to an azimuthal rotation of its eight neighbours by 10° as determined by a inspection of the intramolecular features. To probe the YSR states, constant height maps of the current at 2 mV, *i.e.* on the low-voltage edge of the YSR peaks, were recorded. In the original array, the centre molecule exhibits a fairly strong YSR signal while the signal is weak on all eight neighbours (Fig. 10(c)), whose YSR states occur at slightly higher voltage. After manipulation (Fig. 10(d)), no YSR state is discernible at the centre molecule, which is now in the ↓ state. However, strong signals have arisen on the four nearest neighbours (edge molecules) and medium signal is found on the next nearest neighbours (corner molecules), reflecting downshifts of their YSR states. At present, a detailed modelling of these striking changes has not been obtained. Our previous results from similar arrays of metal-free phthalocyanine on Pb(100) suggest that electrostatic interactions *via* induced charge densities most likely play an important role.^54^ In any event, the data show that artificial spin arrays can be made from InPc and that they exhibit a degree of tunability.

## 6 Discussion

Charge transfer between organic adsorbates and solid substrates is a ubiquitous effect that leads to changes of the physico-chemical properties, the spin state being no exception.^55–60^ For this reason, the spin state switching reported here is not unexpected. In the present case, the distance of the molecular centre from the Pb(100) substrate determines the spin state of the complex. A presumably related effect has been reported from a number of molecules whose spin state or magnetic anisotropy can be modified by bringing the tip of the STM closer to the molecule.^34,61–67^

We hint that other molecules that exhibit geometric bi-stability at surfaces may similarly undergo a change of their spin state when a geometric transition takes place. Indeed, preliminary measurements of SnPc on Pb seem to confirm this hypothesis.^50^ This raises the intriguing question whether the switching of SnPc know from Ag substrates may actually also involve a spin change. According to a theoretical study, the inversion of SnPc involves a reversible two electron oxidation and intersystem crossing.^68^

It will be interesting to investigate other bi-stable complexes for spin switching. The use of YSR states as spin state fingerprints is unfortunately limited to superconducting substrates. While the Kondo effect has already served to discover spin state switching of retinoic acid,^69,70^ methods like spin-polarized STM,^71,72^ shot noise measurements^73,74^ and possibly optical spectroscopies^75,76^ may be viable alternatives to address other substrates.

## 7 Conclusions

InPc on Pb(100) was investigated with low-temperature scanning tunnelling microscopy. Isolated molecules as well as molecules in ordered arrays were prepared in two distinct states with in either below or above the macrocycle. Switching from InPc↑ to InPc↓ was demonstrated. The InPc↑ molecules in islands carry a localized spin as reflected by YSR states and have controllably been switched to a non-magnetic ↓ state. The data show that the combination of charge transfer at surfaces with mechanically bi-stable molecules may serve to develop a new class of magnetic switches.

## Data availability

The data used are available from the authors upon reasonable request.

## Conflicts of interest

There are no conflicts to declare.

## Supplementary Material

RA-014-D4RA07270G-s001
