# Supplementary material for: Spin-state switching of indium-phthalocyanine on Pb(100)
Source: RSC Adv. 2024 Dec 5;14(52):38506–13. doi: 10.1039/d4ra07270g (PMC11618534; doi:10.1039/d4ra07270g)
Supplement: RA-014-D4RA07270G-s001 [file RA-014-D4RA07270G-s001.pdf]

Supplementary Information (SI) for RSC Advances.
This journal is © The Royal Society of Chemistry 2024

Supporting Information: Spin-State Switching of Indium-Pthalocyanine on Pb(100)

Niklas Ide, Arnab Banerjee, Alexander Weismann, and Richard Berndt*

Institut für Experimentelle und Angewandte Physik,
Christian-Albrechts-Universität, 24098 Kiel, Germany

E-mail: berndt@physik.uni-kiel.de

Adsorption Site Determination

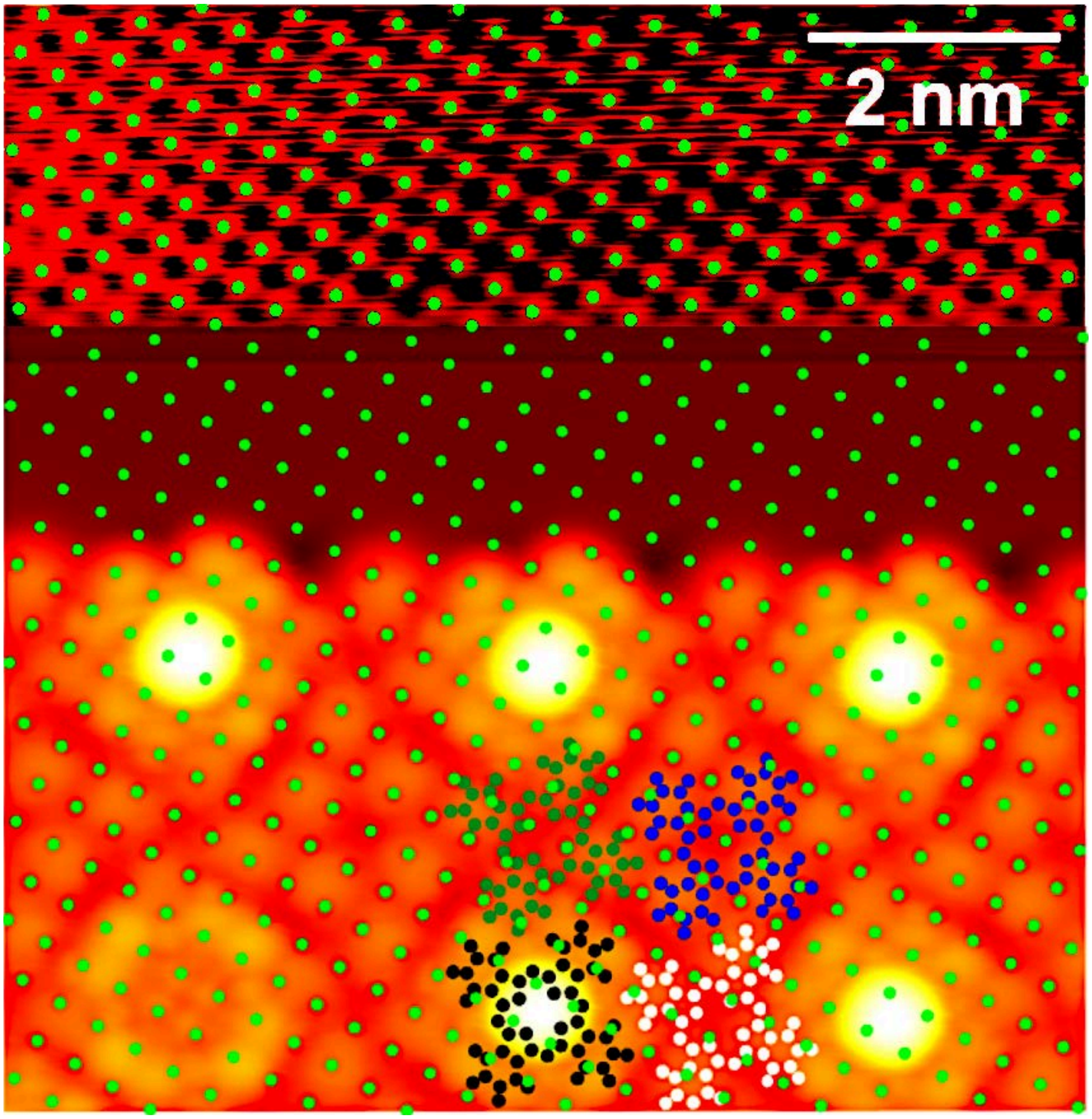

Figure S1: Topograph ($V = 10\,\mathrm{mV}$) of an ordered island of InPc molecules. The current setpoint was $I = 5\,\mathrm{nA}$ in the upper part of the image, where the substrate atoms are resolved. The Pb(100) lattice is marked by green dots and has been extrapolated to the bottom part of the image. The current was lowered to $100\,\mathrm{pA}$ in the lower part to image the molecular layer. Four adsorption sites (top, bridge x, hollow, bridge y) have been identified and are indicated by blue, green, black, and white models of InPc molecules. The color scales used in the upper an lower parts of the image have been separately adjusted.

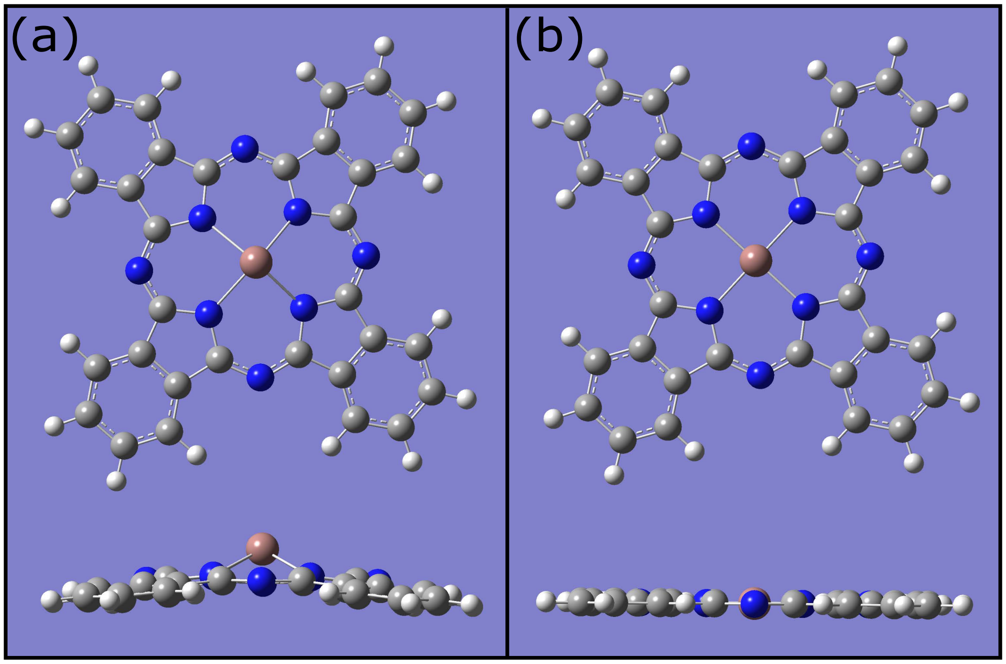

Figure S2: DFT-optimized structures of (a) neutral and (b) singly positively charged InPc. The neutral InPc molecule displays a shuttlecock-like shape while the charged molecule is planar.
